# Parental supervision, children’s self-control and smartphone dependence in rural children: a qualitative comparative analysis from China

**DOI:** 10.3389/fpsyg.2025.1481013

**Published:** 2025-03-31

**Authors:** Na Li, Wushuang Liu, Suzhen Yu, Rui Yang

**Affiliations:** ^1^Department of Sociology, School of Humanities and Social Sciences, Xi’an Jiaotong University, Xi’an, China; ^2^Department of Public Administration, School of Humanities, Chang’an University, Xi’an, China

**Keywords:** rural children, China, smartphone dependence, qualitative comparative analysis, parental supervision

## Abstract

**Objective:**

This study explored the developmental pathways of smartphone dependence among rural children in China, focusing on the interplay between parental supervision, children’s self-control, and parent–child relationships.

**Methods:**

In-depth interviews were conducted with 20 rural Chinese children and their parents. A Qualitative Comparative Analysis was employed to examine the conditions and mechanisms underlying smartphone dependence from both children’s and parents’ perspectives.

**Results:**

Three distinct pathways to smartphone dependence were identified. Path 1: Children who were not left behind exhibited low self-control, lacked supervision and guidance, and had introverted personalities, and were more susceptible to smartphone dependence. Paths 2 and 3— Children who were left behind in rural areas and lack self-control were prone to developing smartphone dependence, regardless of whether they had a distant or harmonious parent–child relationship and an introverted or extroverted personality.

**Conclusion:**

Children’s self-control and parental supervision were critical factors influencing the participants’ smartphone dependence. The children’s sex, age, academic performance, parents’ smartphone use duration, and primary caregivers’ parenting skills moderated these influencing paths. Interventions should focus on enhancing children’s self-control through skill-building and equipping parents and primary caregivers with effective supervision, communication, and boundary-setting strategies to foster healthier technological habits.

## 1 Introduction

Smartphones are essential communication tools that encompass functions such as social contact, information transmission, resource acquisition, entertainment, and leisure (Elhai et al., 2017). With the continuous development of the internet and optimization of smartphone functionalities, the number of smartphone users has expanded spatially and across various age groups. This influence has gradually permeated children’s lives, leading to widespread smartphone dependence. In rural China, the scarcity of recreational facilities limits children’s access to diverse leisure activities during their free time. Concurrently, rapid urbanization has led to the significant migration of rural parents to urban areas for employment, resulting in widespread parental absence and inadequate supervision of rural children. The population of left-behind children in rural China is significant, exceeding 61 million in recent years (Shan et al., 2023; Zhang et al., 2023; Zhen et al., 2019). These children face unique challenges due to the separation from their parents, which often leads to emotional difficulties such as loneliness and depression, as well as issues related to their academic engagement and mental health (Elhai et al., 2017; Troll et al., 2020; Zhang et al., 2023). Parental neglect or absence in rural areas creates considerable strain on parent–child relationships. Although many migrant parents try to maintain communication through smartphones, the emotional and behavioral support that their children require from their parents is often lacking. The challenges of parental supervision are compounded by the limited education levels of grandparents, who often lack the skills to effectively manage their grandchildren’s emotional and behavioral needs (He et al., 2022). Given these factors, smartphone dependence among rural children is becoming increasingly severe. A research team survey in the central provinces of China revealed that approximately 90% of left-behind rural children regularly use their own or their guardians’ smartphones for entertainment (Zhu and Lei, 2023). This underscores the prevalence of smartphone dependence in rural areas and highlights the urgency of addressing this issue.

Smartphone overuse is significantly associated with academic problems (Troll et al., 2020; Zhang et al., 2023), depression, anxiety (Elhai et al., 2017), internet overuse, alcohol dependence, nicotine dependence, myopia, and other health risks (Bae, 2024; Jo et al., 2021; He et al., 2022). Given that smartphone dependence not only threatens the physical and mental health of rural children, but also poses significant risks to family relationships and social stability, extensive research has been conducted on this phenomenon. Previous studies have primarily focused on children and their families, revealing that individual smartphone dependence is largely influenced by personal factors such as personality traits (e.g., the Big Five personality traits, including neuroticism, extraversion, openness, agreeableness, and conscientiousness) (Cocorada et al., 2018; Horwood and Anglim, 2018), capacity for self-control (involving the conscious suppression of undesirable behaviors, self-monitoring, and restraining automatic responses) (Muraven and Baumeister, 2000; Sok et al., 2019), and parental characteristics at the family level (e.g., the family’s socioeconomic status) (Xiao et al., 2024). These behaviors have been shown to negatively affect individuals’ physical and mental health, character development, and social interactions (Karaca et al., 2020; Li and Guo, 2016). However, prior studies have not thoroughly analyzed the combined characteristics of parents and children within a comprehensive framework to better understand the multiple pathways that lead to children’s smartphone dependence. Moreover, few studies have offered practical, theory-based intervention measures to address this issue. By exploring the formation mechanisms of rural children’s smartphone dependence and potential solutions, this study aims to deepen understanding of the issues among scholars and practitioners while providing valuable insights for addressing smartphone dependence among rural children.

## 2 Literature review

### 2.1 Smartphone dependence

Scholars have approached the concept of smartphone dependence from various perspectives, including as a pathological condition, an impulsive and uncontrolled behavior, an emotional dependence disorder, as well as other categories, resulting in significant variations in results (Sohn et al., 2019). Despite these differences, there is general agreement regarding the core aspects of smartphone dependence. It is commonly understood as the excessive use of smartphones which can negatively impact an individual’s physical and psychological well-being, leading to the loss of certain social functions.

Park (2019) categorized smartphone dependence into two types: functional dependence, where users rely on smartphones for tasks such as acquiring news or scheduling, and existential dependence, which is characterized by compulsive emotional attachment and habitual checking of social media for reassurance. The study found that users with existential dependence were less likely to recognize the negative aspects of their behavior and were more resistant to change, whereas those with functional dependence were more open to altering their habits.

This study will incorporate the conceptual definition of smartphone dependence proposed by Wang et al. (2017), which differentiates smartphone addiction from internet addiction: Smartphone dependence is defined as a novel form of addictive behavior characterized by excessive smartphone usage, an inability to regulate one’s own smartphone use, and subsequent impairment of social functioning as well as psychological and behavioral issues for the individual.

### 2.2 Individual factors and smartphone dependence

Academic research on smartphone dependence began in 2001 (Wu, 2018). Subsequent studies, building on earlier analyses, have focused primarily on identifying the individual and familial factors contributing to smartphone dependence.

Smartphone-dependent behavior is primarily predicted by an individual’s own characteristics including personality traits and capacity for self-control. Horwood and Anglim (2018) used the broad and narrow traits of the HEXACO and Five Factor Models of personality to predict problematic smartphone use. Research suggests that individuals with low levels of security, low self-esteem, and high levels of loneliness are more likely to immerse themselves in the online world, developing a greater need for smartphone usage, which, in turn, intensifies their dependence on smartphones (Horwood and Anglim, 2018; Elhai et al., 2017). One notable theory is the compensatory internet use theory, which posits that individuals turn to the internet and smartphones to escape real-life problems or compensate for failed situations. In this context, introversion and social anxiety are common motivators (Horwood and Anglim, 2018; Kardefelt-Winther, 2014).

Smartphones can substitute virtual socialization for face-to-face interactions, thus alleviating the anxiety and fear commonly experienced by introverts during social encounters. It also offers a way to escape reality or seek self-compensation through participation in virtual communities, such as gaming platforms or social forums (Parent et al., 2021). Liu et al. (2023) emphasized the importance of trait mindfulness as a psychological resource that could reduce the risks associated with smartphone dependence, especially for vulnerable groups such as left-behind children.

Smartphone dependence is also influenced by individuals’ capacity for self-control. Self-control is the ability to consciously suppress undesirable actions, monitor one’s own behavior, and restrain automatic responses (Sok et al., 2019; Troll et al., 2020). Although self-control theory is typically applied in criminology (Gottfredson and Hirschi, 1990), other studies have shown that self-control has strong explanatory power for common problematic behaviors. Low self-control is closely associated with behaviors such as internet deviance and addiction (Liu et al., 2022; Sok et al., 2019). Self-control has also been identified as a mediator in the relationship between social exclusion and smartphone addiction. Individuals with lower levels of self-control are more prone to excessive smartphone use (Sun and Yoon, 2023), particularly when experiencing social exclusion (Yue et al., 2022). Zhang et al. (2019) found that individuals with higher levels of self-control were less likely to develop smartphone dependence even when their interpersonal adaptation skills were low.

### 2.3 Family factors and smartphone dependence

Family is the primary domain of children’s lives, and parents play a crucial role in shaping their growth and development. According to the family systems theory proposed by Bowen (1978), the family functions as a stable system collectively constructed by all its members. Within this system, specific patterns of communication exist among family members, leading to various interactions in daily life. According to this theory, as rural children are not autonomous individuals but rather family members reliant on their familial environment for survival, families may serve as both the instigators of children’s smartphone dependence behavior and a potential avenue for mitigating it.

Previous studies have provided valuable insights into the predictive paths of family system factors on smartphone dependence. Factors such as family parenting style and parent–child relationships impact an individual’s propensity for smartphone dependence. Parenting style, a crucial form of daily interaction between parents and their children, significantly influences the process of smartphone acquisition, usage patterns, addiction tendencies, and other aspects related to smartphone dependence. As a result, different parenting styles yield distinct outcomes in terms of smartphone dependence. Specifically, an authoritarian parenting style characterized by excessive protection and psychological control increases the likelihood of children developing smartphone dependence. Conversely, an understanding parenting style marked by emotional warmth from parents can effectively reduce children’s dependent behaviors associated with smartphone use (Lian et al., 2016; Li et al., 2021).

Supervision (the amount and quality of time parents spend monitoring their children’s activities) is a key component of parenting style. It includes both passive supervision (being physically present) and active engagement (participating in activities with the child). Effective supervision helps ensure that adolescents adhere to family rules and limits, thereby reducing the likelihood of engaging in risky or problematic behaviors, particularly in disadvantaged socioeconomic environments (Hayes et al., 2004; See, 2014; Wilson, 1980). Parents with different parenting styles provide varying levels of parental supervision over their children’s smartphone use. If parents establish effective family rules and children know about the monitoring (Fischer, 1983), the children are more likely to adhere to parental guidance and reduce smartphone usage, even in the absence of parental presence due to work-related travel (Laird et al., 2009). The parent–child relationship is also a crucial reflection of the family environment, since parental supervision indirectly impacts these outcomes through family support. The quality of the parent–child relationship, marked by emotional connectedness and effective communication, has been shown to buffer the negative impact of adversity on these children (West et al., 2021). Shao et al. discovered a significant negative correlation between poor parent–child relationships and children’s dependence on smartphones (Shao et al., 2024). Chen et al. (2023) examined how discrepancies between adolescents’ and parents’ perceptions of parental phubbing—defined as parents prioritizing smartphone use over engaging with their children, which leads to neglect of face-to-face communication—contribute to adolescent smartphone dependence, with the parent–child relationship serving as a mediator in this process. This suggests that improving communication between parents and children can help reduce smartphone addiction. Establishing and strengthening a healthy parent–child relationship could effectively regulate children’s duration of smartphone usage, thereby reducing the likelihood of excessive use. However, Zhen et al. (2019) found that parent–child relationships did not buffer the impact of negative life events on smartphone dependence.

Social learning theory also offers valuable insights. According to this theory, individuals acquire social behaviors predominantly through observation and imitation (Banerjee, 1992). Childhood is an important stage in individual cognitive shaping and ideological development; therefore, children are more inclined to seek learning standards and experiential knowledge from communication with their parents. Therefore, rural children’s smartphone dependence is largely a product of imitation, which is a copy of their parents’ smartphone use behavior. In addition, under the influence of family media use habits, the parents’ dependence on smartphones will be transferred from parents to children, worsening children’s interactive experiences, harming their mental health, and distorting their cognition of smartphone use (Chen, 2023).

### 2.4 Strategies to mitigate rural children’s smartphone dependence

In recent years, rural left-behind children, as a vulnerable group, have increasingly become the focus of local governments and social work agencies. Currently, these agencies conduct intervention studies on rural children in disadvantaged families to ensure their protection and security, and professional methods are employed to provide targeted support for children based on their specific circumstances (Green and Ayalon, 2020). Furthermore, there has been a growing body of research focused on the promotion of positive and comprehensive development among rural children. Multi-faceted intervention studies have been conducted on cultural education, life education and growth and development (Pečnik et al., 2024; Philip, 2015). However, only a limited number of intervention studies have been conducted on smartphone dependence among rural children. Currently, intervention research on this issue is primarily theoretical and predominantly focused on identifying problems rather than exploring the specific developmental and optimization paths that align with the unique characteristics of rural children (White et al., 2019). Furthermore, few intervention studies related to smartphone dependence among rural children have systematically and scientifically analyzed the formation mechanisms of such dependence. Consequently, accurately identifying the specific leverage points for interventions becomes challenging, leading to a significant reduction in the effectiveness of such programs.

### 2.5 Limitations of previous studies

There remains significant potential for expanding research in several key areas. First, most existing studies on smartphone dependence have primarily adopted psychological or communication perspectives, concentrating extensively on individual factors such as personality traits, self-control, and external media influences, often overlooking the critical role of family dynamics in smartphone dependence development (Chen et al., 2023; West et al., 2021; Zhen et al., 2019). Additionally, prior research has largely focused on college students, who typically have greater autonomy regarding smartphone usage (Park, 2019; West et al., 2021); however, childhood represents a crucial developmental period that necessitates positive societal and familial guidance, particularly for vulnerable populations such as left-behind children in rural areas (Shan et al., 2023; Zhen et al., 2019). Methodologically, most studies have primarily relied on traditional quantitative methods, such as regression and correlation analyses, alongside qualitative approaches including interviews and ethnographic techniques (Schneider and Wagemann, 2012). Nevertheless, there has been limited examination of multiple causal pathways and the underlying mechanisms contributing to smartphone dependence. Consequently, there is a need for more comprehensive methodological frameworks, such as Qualitative Comparative Analysis (QCA), capable of capturing complex causal configurations and nuanced case-level differences (Ragin, 1987; Schneider and Wagemann, 2012). Finally, while many researchers have emphasized theoretical models explaining smartphone dependence (Chen et al., 2023; Shan et al., 2023), relatively few studies provide practical, empirically-informed intervention strategies tailored specifically to the Chinese rural context. Therefore, significant opportunities remain to develop actionable, empirically grounded interventions aimed at effectively addressing smartphone dependence among rural children.

Based on this, we employed QCA to investigate the formation mechanism and adverse effects of smartphone dependence among rural children, focusing on the two key dimensions of children and their parents. Based on the analysis results and considering the physical and mental immaturity of children, this study formulated specific intervention strategies from the perspective of parents and primary caregivers to help rural children form correct cognition of smartphones, solve the problem of smartphone dependence, and help rural children’s healthy growth and positive development.

## 3 Research design

### 3.1 Methods

Qualitative Comparative Analysis (QCA), a methodology specifically developed to investigate complex causality within small to medium-sized samples (Ragin, 1987; Schneider and Wagemann, 2012), was employed in this study. By emphasizing detailed comparative analyses at the case level, QCA systematically identifies distinct configurations of conditions associated with particular outcomes through the construction of truth tables (Ragin, 2008). Even with relatively few cases, QCA effectively captures conjunctural causation, uncovering multiple causal pathways (equifinality) leading to the same outcome (Schneider and Wagemann, 2012). Additionally, this approach enables researchers to explore causal complexity by identifying necessary and sufficient conditions through systematic case comparisons (Thomann and Maggetti, 2020). As a qualitative methodology, QCA supports inductive analyses, utilizing data from interviews, secondary sources, and other qualitative materials, thus providing comprehensive insights into the underlying mechanisms of smartphone dependence among rural children (Marx et al., 2014). This approach allowed for a systematic investigation of factors contributing to rural children’s smartphone dependence behavior, the interactions among these factors, and possible combinations of these factors, thus enabling a more effective exploration of the complex causes of smartphone dependence. Overall, QCA effectively supplements methods used in previous studies while offering more abundant and profound explanations to deepen our understanding of the mechanisms underlying the formation of smartphone dependence among rural children. In particular, crisp-set QCA (csQCA) was adopted because most of the variables were binary.

### 3.2 Data and sample

According to Marx and Duşa’s (2011) simulation analysis, the number of cases and conditions significantly influences the consistency solutions obtained through QCA. They provided benchmarks indicating the minimum number of cases required for different numbers of conditions; for instance, a QCA model with four conditions should include at least 12 cases, while a model with five conditions requires at least 15 cases. As the number of conditions increases, the required number of cases correspondingly rises. Therefore, careful consideration of the required number of cases is necessary during the research design stage to ensure robust QCA results. Twenty rural children from 11 provinces in China (Jilin, Liaoning, Hebei, Tianjin, Henan, Anhui, Zhejiang, Jiangxi, Fujian, Guangxi, and Yunnan) were recruited online through social media platforms via online publicity and voluntary registration. To comprehensively assess smartphone dependence among rural children and mitigate biases arising from sample selection while analyzing the role of parental supervision in the development of children’s smartphone dependence, this study included both left-behind and non-left-behind rural children within its scope. Although previous studies have offered varying definitions for left-behind children, the academic community generally agrees that “parental migration” is the core element of this concept, meaning that rural left-behind children are those with one or both parents working away from home (Pan and Ye, 2009). In this study, to understand the unique impact of total parental absence on children’s smartphone dependence and to enhance the focus and scientific rigor of the research, children with both parents working away from home for more than half a year were defined as left-behind children, while those with only one or neither parent away were categorized as non-left-behind children.

The demographic details of the rural child participants are presented in Table 1. There are 13 left-behind children with both parents working away and 7 non-left-behind children. To assess smartphone dependence, we used a self-report questionnaire and conducted in-depth interviews using a customized interview guide. The self-reported questionnaire, titled the *Adolescent Smartphone Use Dependency Self-Assessment Questionnaire*, was developed by Tao et al. (2013), and has been validated through confirmatory factor analysis, correlation analysis, and internal consistency tests, demonstrating satisfactory reliability and validity in accordance with psychometric standards.

**Table 1:** Rural child respondents’ demographic information.

No.	Sex	Age	Location	Smartphone possession	Left-behind children	Measurement score	Smartphone dependence
1	F	9	D Village, Hebei	No	Yes	40	No
2	F	14	H Village, Anhui	Yes	Yes	42	No
3	F	15	J Village, Jiangxi	Yes	Yes	57	Yes
4	M	7	H Village, Henan	Yes	Yes	51	Yes
5	F	15	Z Village, Zhejiang	Yes	Yes	50	Yes
6	M	13	D Village, Fujian	Yes	Yes	52	Yes
7	F	13	D Village, Henan	Yes	Yes	37	No
8	F	9	H Village, Tianjin	Yes	Yes	49	Yes
9	F	8	H Village, Henan	Yes	Yes	42	No
10	F	15	H Village, Henan	Yes	Yes	51	Yes
11	M	12	L Village, Liaoning	Yes	Yes	26	No
12	M	10	L Village, Liaoning	Yes	Yes	27	No
13	M	12	J Village, Jiangxi	No	Yes	36	No
14	M	11	H Village, Hebei	Yes	No	41	No
15	M	12	H Village, Hebei	Yes	No	43	No
16	M	15	H Village, Henan	Yes	No	55	Yes
17	F	13	F Village, Fujian	Yes	No	45	No
18	M	13	Y Village, Yunnan	Yes	No	50	Yes
19	M	12	J Village, Jilin	Yes	No	27	No
20	M	9	B Village, Yunnan	No	No	49	Yes

The questionnaire comprises three dimensions: withdrawal symptoms, craving for smartphones, and physical and psychological impacts. Each dimension includes five items rated on a scale from 1 to 5, indicating increasing severity. The scores for all 13 items were summed for each participant. Following the guidelines provided by the questionnaire’s developers, if a child’s total score exceeded 75% of the maximum possible score for all questions combined, it indicated the presence of smartphone-dependence. Furthermore, higher total scores reflected a greater degree of smartphone dependence. The measurement results revealed that nine children scored above the cutoff point of 48.75, indicating smartphone dependence, while eleven children scored below this threshold.

### 3.3 Selection and settings of variables

The selection of variables is a critical component of the csQCA, as it directly impacts the accuracy and scientific rigor of the research findings. We adopted a two-dimensional approach (considering both parents and children) to select conditional variables. Five variables were included in the analysis: parental migration for work more than half a year (left-behind children), quality of parental supervision and guidance, parent–child relationship quality, children’s capacity for self-control, and children’s personality traits. The definitions and references of these variables are presented in Table 2.

**Table 2:** Definitions and references of variables.

Variables	Definitions of variables	References and criteria
Smartphone dependence	Smartphone dependence refers to a novel form of addictive behavior characterized by excessive smartphone usage, an inability to regulate one’s own smartphone use, and subsequent impairment in social functioning as well as psychological and behavioral issues for the individual.	Measure by the scale developed by Tao et al. (2013)
Left-behind children	Left-behind children are those living in rural areas of China, usually raised by grandparents or other relatives, while their parents have migrated to urban areas for work more than half a year.	He et al. (2022) and Zhang et al. (2023)
Parental supervision	Parental supervision refers to the time parents spend monitoring their children’s activities and the quality of that time, encompassing both passive supervision (being physically present) and active engagement (participating in activities with the child).	Fischer (1983), Hayes et al. (2004), Roberts (1996), See (2014), and Wilson (1980)
Parent–child relationship	The quality of the parent–child relationship particularly refers to parental warmth, trust, and the ability of adolescents to disclose personal matters.	He et al. (2022), Melton and Deutsch (2020), West et al. (2021), and Zhen et al. (2019)
Self-control	Self-control is the ability to suppress undesirable actions, monitor behavior, and restrain automatic responses.	Sok et al. (2019), Troll et al. (2020), and Zhang et al. (2019)
Personality traits	Personality traits are defined as a set of broad and narrow traits that shape how individuals respond to their environment and manage different situations.	Horwood and Anglim (2018)

As shown in Table 2, we designed the interview outline based on the definitions of each variable and coded the variables based on the responses of both children and parents. At the parental level, when rural parents work in urban areas and entrust their children to grandparents, left-behind children experience a prolonged absence of parental emotional support, supervision, and guidance. This lack of parental presence makes them more likely to experience feelings of loneliness and emptiness, leading them to seek solace in the virtual world of the internet, thereby intensifying their reliance on smartphones.

Conversely, when parents adopt positive methods for supervising and guiding their children, such as establishing structured routines and plans for daily life, and assisting in cultivating good habits, they enhance their children’s self-discipline and awareness of rules. This then indirectly strengthens their resistance to the temptation of using smartphones. The act of parents leaving home, along with its associated loss of supervision and guidance, plays a pivotal role in shaping phone-dependent behavior patterns. Therefore, we incorporated two variables: parental migration for work and quality of parental supervision and guidance. Furthermore, the specific impact of parental supervision and guidance is also influenced by parent–child relationships. A harmonious parent–child relationship fosters greater compliance with parental discipline and reinforces efforts aimed at addressing smartphone dependence. Since both parental supervision/guidance and a harmonious parent–child relationship are core influencing factors, the parent–child relationship was also selected as a conditional variable in the analysis.

At the child level, existing research indicates that low self-control in children reduces their resistance to the allure of smartphones, leading to internet addiction and an escalating dependence on smartphones. Additionally, smartphones equipped with screen isolation features and the anonymity provided by the internet provide a more private and less restrictive environment for self-expression compared to the real world, which further deepens individual reliance on smartphones. Considering these factors, we selected children’s self-control and personality as conditioning variables for the analysis.

The outcome variable was the assessment of rural children’s smartphone dependence, which involved categorizing them into dependent and non-dependent groups based on their scores on a self-assessment questionnaire on smartphone dependence.

The condition and outcome variables were transformed into binary variables with a value of 0 or 1, as specified in Table 3. The descriptions of the conditional variables were derived from interviews with 20 rural children and assigned corresponding values: for parental factors, having both parents migrate for work was coded as 1, while having only one parent doing so was coded as 0; presence of parental supervision and guidance was coded as 1, while the lack thereof was coded as 0; a harmonious parent–child relationship was coded as 1, while estrangement between parent and child was coded as 0. Regarding child-related factors, strong capacity for self-control was given a value of 1, whereas poor self-control received a value of 0; an extroverted personality in children was coded 1, whereas an introverted personality received a code of 0. Regarding the outcome variable, phone dependence was coded as 1, while phone independence was coded as 0. The coding rules and statistical results are presented in Table 3.

**Table 3:** Variable selection and settings.

Variable	Variable name	Code	Judgment criteria	Assignment	Number	Proportion
Conditions	Parental migration for work	LB	Both parents are migrated	1	13	65.00%
			One parent is migrated	0	7	35.00%
	Parental supervision and guidance	GUID	Parental supervision	1	9	45.00%
			Parental neglection	0	11	55.00%
	Parent–child relationship	PR	Harmonious parent–child relationship	1	13	65.00%
			Estranged parent–child relationship	0	7	35.00%
	Self-control in children	CTRL	Children have strong self-control	1	6	30.00%
			Children have poor self-control	0	14	70.00%
	Children’s personality	CHAR	Extroversion	1	7	35.00%
			Introversion	0	13	65.00%
Outcome	Children’s smartphone dependence	MPADD	Smartphone dependence	1	9	45.00%
			Smartphone independent	0	11	55.00%

## 4 Results

The fsQCA3.0 software was used to conduct a qualitative comparison analysis of crisp set data. The analysis procedure was as follows. First, the value table was constructed, and a Venn diagram was drawn to observe the distribution of sample configurations and their proportional representation. The contradictory configurations were checked and resolved. Second, based on the truth table, a Boolean minimization operation was performed to derive all conditions or configurations of the outcome were obtained along with their coverage and consistency. Additionally, the coverage and consistency of solutions are also determined. A condition or configuration was considered a sufficient condition for the outcome if its consistency score exceeded 0.85. A condition or configuration was regarded as a necessary condition if it always occurred when the outcome happened (Rihoux and Ragin, 2009). Consistency contributes to theory development, but does not empirically determine the extent to which a condition or configuration explains an outcome (Pennell et al., 2013). In contrast, coverage measures how well the set that passes the consistency test explains the result, reflecting its empirical relevance in explaining the outcomes. The higher the coverage value, the stronger the explanatory power of the configurations in relation to the results (Rihoux and Ragin, 2009). Finally, the results of the analysis were organized and interpreted.

**Figure 1:**
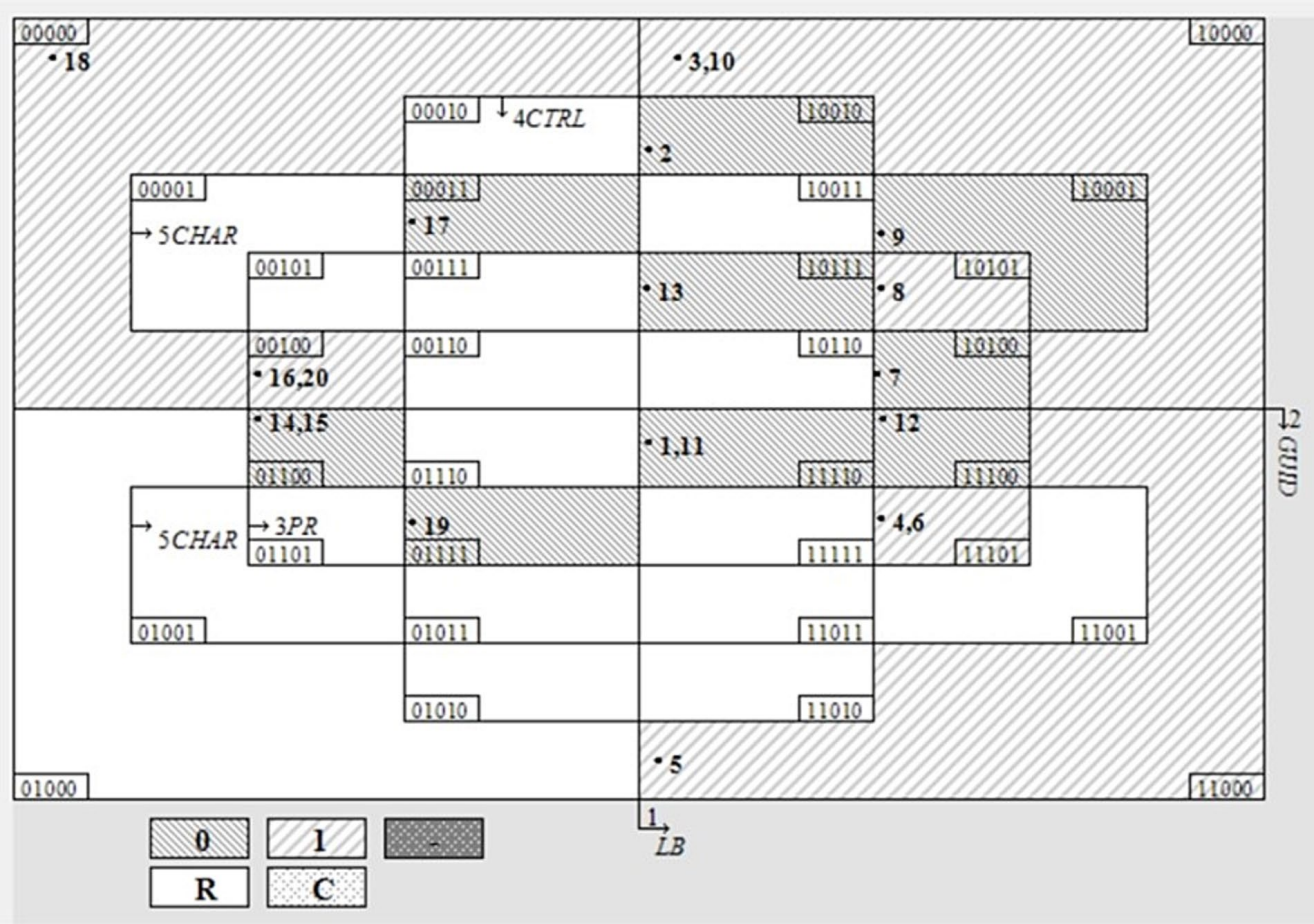
Venn diagram.

The truth table is presented in Table 4, and Figure 1 illustrates the Venn diagram of the configuration distribution. In this diagram, “0” denotes the outcome variable “smartphone independent,” and “1” represents “smartphone dependent.” “R” signifies the logical remainder, which refers to configurations not covered by the truth table. “C” indicates contradictory configurations where different outcomes occur under identical conditions. Additionally, the symbol “-” represents missing values.

**Table 4:** Truth table.

Case ID	LB	GUID	PR	CTRL	CHAR	MPADD
18	0	0	0	0	0	1
17	0	0	0	1	1	0
16, 20	0	0	1	0	0	1
14, 15	0	1	1	0	0	0
19	0	1	1	1	1	0
3, 10	1	0	0	0	0	1
9	1	0	0	0	1	0
2	1	0	0	1	0	0
7	1	0	1	0	0	0
8	1	0	1	0	1	1
13	1	0	1	1	1	0
5	1	1	0	0	0	1
12	1	1	1	0	0	0
4, 6	1	1	1	0	1	1
1, 11	1	1	1	1	0	0

Figure 1 presents the Venn diagram used in QCA, visually depicting the relationships among different causal conditions and their configurations (Schneider and Wagemann, 2012). Each distinct area in the diagram represents a unique combination of conditions (coded as binary digits), with shaded and unshaded regions indicating the presence (“1”) or absence (“0”) of the outcome. Numeric labels within each area correspond to specific cases or configurations. By examining the distribution and frequency of cases across these regions, researchers can systematically identify configurations of conditions that consistently lead to the studied outcome, facilitating the interpretation of complex causal patterns. The Venn diagram demonstrates that the sample encompassed 46.88% of all configurations, with a relatively high coverage rate and no observed contradictions or missing values.

The results of the necessary sufficient condition analysis are presented in Table 5. The findings support the notion that intermediate solutions provide better explanations than parsimonious and complex solutions, as suggested by previous studies (Fiss, 2011; Rihoux and Ragin, 2009). In a configuration, an uppercase letter denotes the presence of a condition (coded as “1”), while a lowercase letter indicates the absence of a condition (coded as “0”). The symbol “*” denotes logical conjunction (“and”), whereas “+” represents logical disjunction (“or”).

**Table 5:** Truth table algebraic analysis results.

Intermediate solution	Raw coverage	Unique coverage	Consistency
lb*ctrl*guid*char	0.33	0.33	1
LB*ctrl*pr*char	0.33	0.33	1
LB*ctrl*PR*CHAR	0.33	0.33	1
Solution coverage	1		
Solution consistency	1		

The results presented in Table 5 demonstrate three distinct paths leading to smartphone dependence among rural children. Both the raw coverage and unique coverage for each path were 0.33, indicating that each path accounted for approximately 33% of the cases and contributed significantly to the overall explanation of smartphone dependence. The consistency value for each path is 1, suggesting that each path alone is sufficient to produce an outcome. Furthermore, the solution consistency also equals 1, confirming the adequacy of all identified paths. Finally, with an overall coverage rate of 1, it can be concluded that the program effectively captures and explains rural children’s level of smartphone dependence.

The findings from Path 1 (lb*ctrl*guid*char, i.e., children with one migrated parent * lack of self-control * lack of supervision and guidance * introverted personality) indicate that the rural non-left-behind children were prone to developing smartphone dependence due to the combined effects of inadequate supervision and guidance, poor self-control, and an introverted personality. The introverts tended to rely on smartphones for communication with friends, relatives, and the outside world. Moreover, they exhibited a stronger preference for online entertainment than offline activities when using their smartphones. In the absence of sufficient self-control and parental oversight, these children were more likely to engage in excessive smartphone use, leading to dependence.

Paths 2 (LB*ctrl*pr*char) and path 3 (LB*ctrl*PR*CHAR) can be expressed as LB*ctrl*(pr*char+PR*CHAR), indicating that left-behind children with poor self-control, were prone to developing smartphone dependence regardless of whether they had distant parent–child relationships and introverted personalities, or harmonious parent–child relationships and extroverted personalities. In Path 2, because both parents were away from home, the children had limited opportunities to interact with their parents. Prolonged low-frequency communication leads to a strained parent–child relationship. Children lack companionship and emotional support and thus seek emotional compensation from smartphones. As their trust and obedience towards their parents decreased, they became less inclined to actively listen to supervision and guidance on smartphone usage or engage in active learning experiences. Additionally, for children with an introverted personality and poor self-control, smartphones have gradually become a significant source of instrumental and emotional support, deepening their dependence on them.

Regarding Path 3, in the case of children with an extroverted personality, poor self-control, frequent contact with parents and a positive parent–child relationship, even when both parents migrate for work, there are potential explanations for their smartphone dependence. First, due to physical distance from their children, parents can only supervise and guide their children’s smartphone usage through video calls, which has limited effectiveness. Second, extroverted children often exhibit less fear of parental supervision and are more receptive to criticism, which further diminishes the impact of parental supervision and guidance. Consequently, when self-control is poor, smartphone dependence is more likely to occur. Additionally, some interview data indicated that parents’ indulgent behavior and leniency towards excessive smartphone use increased children’s dependence on smartphones.

A lack of self-control (ctrl) was evident in all three scenarios, indicating that it is a necessary condition for rural children’s reliance on smartphones. This finding is consistent with that of West et al. (2021), who identified self-control as the only unique predictor of smartphone dependence even after considering reward processing and depressive symptoms. The less self-control children possess, the more reliant they become on their smartphones. This is partly attributable to children’s incomplete psychological development, resulting in a relatively low ability to discern right from wrong, restrain themselves, and resist external temptations. During smartphone usage, it becomes challenging for children to assess the reasonableness of their phone use or to evaluate whether the information transmitted through mobile devices is self-serving. Moreover, exercising strict self-restraint becomes even more difficult for them, leading to an increased susceptibility to smartphone addiction. Furthermore, with the advancement of big data technology, mobile applications often push a substantial amount of incentivized content to enhance user engagement. Consequently, children with limited self-control are more prone to falling into this trap and developing smartphone dependence.

The overall impact of parental migration, parental supervision and guidance, parent–child relationships, children’s self-control, and children’s personality on rural children’s smartphone dependence behavior is significant. Among these factors, the lack of self-control in children plays a crucial role.

## 5 Discussion

Smartphones played a pivotal role for rural children in addressing everyday challenges, alleviating negative emotions, and facilitating social support (Huang et al., 2023). However, the findings from the case interviews and analysis presented in this study demonstrated that the rural children were susceptible to smartphone dependence, with self-control emerging as a crucial contributing factor. The children with poor self-control were more susceptible to developing smartphone dependence, whereas those with stronger self-control exhibited conscious and rational time management, task prioritization, and established behavioral habits that made them more resilient against the allure of smartphones and less likely to develop dependence. This finding is consistent with previous studies across different age groups and cultural contexts that self-control is a significant predictor of individual smartphone dependence. This reinforces self-control as a universal predictor of smartphone dependence (Jeong et al., 2020; Kim et al., 2018; Li et al., 2016).

Beyond individual factors, parenting practices also played a critical role. Disciplinary methods employed by parents and a failure to provide timely effective communication and positive guidance on smartphone usage can contribute to parent–child alienation, introversion in children, and a lack of normal social interaction. These conditions further exacerbate children’s inclination towards excessive internet use and subsequent development of smartphone dependence. Effective parental supervision played a critical role in preventing or mitigating smartphone dependence in the children. Parental involvement helped to set boundaries and establish guidelines for smartphone use, which can reduce excessive usage and its negative consequences. For instance, setting time limits, monitoring content, and ensuring that children use smartphones for constructive purposes rather than for entertainment alone have been shown to reduce smartphone dependency (He et al., 2022; Zhen et al., 2019).

This study identified the moderating factors of the paths to smartphone dependence in rural children. First, no significant sex differences were found in the influence the paths, with both boys and girls are distributed across these three paths to smartphone dependence, and similarly affected by intrinsic motivations, such as entertainment, socializing, and alleviating negative emotions. This contrasts with the findings of Chen et al. (2017), who concluded that sex moderates these effects, with girls being more influenced by intrinsic motives (e.g., enjoyment and mood regulation) and boys being more influenced by external motives (e.g., social relationships and conformity).

Second, age played a notable role in smartphone dependence, with children aged over 12 being more likely to develop dependence. These children tended to experience communication difficulties with their parents or primary caregivers. In the absence of effective parenting strategies and due to limited self-control, they tended to socialize less offline and rely more on online interactions for social connections, whereas children aged 12 and below maintained a balance between online and offline interactions. Despite peer pressure, younger children engaged in more face-to-face socialization. Therefore, children above 12 required more guidance from parents or primary caregivers to foster healthy offline social interactions, particularly for those who are addicted to online games.

Third, children with average or poor academic performance seemed more prone to smartphone dependence, for they tended to primarily use smartphones for entertainment and social interaction, whereas children with above-average academic performance used their smartphones for entertainment and learning simultaneously. This difference is possibly due to a lack of motivation to learn, insufficient parental supervision, and strained parent–child relationships, with smartphones as a means to escape academic pressure.

Furthermore, the duration of parents’ smartphone usage might serve as a moderating factor in children’s smartphone dependence. In Path 1, the purpose of parents’ smartphone use was particularly crucial: if the usage was work-related, such as responding to work-related messages, its impact on children tended to be minimal. Conversely, excessive use for recreational purposes might have a detrimental effect on children. This finding further validates the conclusions of Park (2019). Regarding Paths 2 and 3, the physical distance between parents and children reduced the influence of parents’ smartphone usage on the smartphone dependence of left-behind children. Social learning theory appears to be less applicable to left-behind children.

Additionally, for left-behind children, the role of other primary caregivers, such as grandparents, was crucial in mediating the effects of lack of parental supervision on smartphone dependence. Even when parents set effective rules, inadequate supervision or negative strategies from primary caregivers (e.g., neglect or punitive measures such as confiscating phones) failed to reduce the children’s dependence. In contrast, reasonable limits and open communication from primary caregivers were associated with lower smartphone dependence among the children. This finding contrasts with that of Shan et al. (2023), who suggested that grandparental support has a delayed positive effect on reducing problematic smartphone use among left-behind children.

Therefore, this paper proposes a comprehensive parental intervention strategy by schools and social work agencies to assist these children in enhancing their accurate comprehension of smartphone usage, rectifying misconceptions, correcting inappropriate behaviors, and cultivating healthy smartphone habits. These strategies require policy and financial support, such as government procurement of social work services in schools or services from social work agencies focused on positive parenting and discipline. Given the large population of rural children, it is crucial to implement changes at the policy level.

As to Path 1, where children who are not left behind exhibit low self-control, lack supervision and guidance, and have an introverted personality, the following intervention strategies could be beneficial. First, Schools and social work agencies should guide parents in setting clear rules and boundaries around smartphone use, such as time limits and appropriate content. Regular discussions about the risks of smartphone dependence and the importance of balance can help parents guide their children’s usage habits. Second, parents can be trained to teach children effective time management and task prioritization. Schools and social work agencies can provide resources or workshops for parents to help children strengthen self-discipline. Introducing self-regulation techniques, such as mindfulness or goal-setting exercises, can also support children’s self-control skills to resist excessive smartphone use. Third, for introverted children, social interaction outside of screen time is crucial. Schools and social work agencies can help parents create opportunities for peer group interactions and extracurricular activities that foster face-to-face communication. These activities help children develop healthier social connections, reducing their reliance on smartphones for emotional fulfillment. Last, schools and social work agencies should encourage parents to model healthy smartphone use by emphasizing the importance of family involvement and limiting their own screen time. Workshops or resources for parents could also promote engaging in family activities that do not involve smartphones, such as games, reading, or cooking together. These practices help build stronger parent–child bonds and reduce smartphone dependence.

For Paths 2 and 3, where children who are left behind in rural areas and lack self-control are prone to smartphone dependence, schools or social work agencies can offer the following interventions for primary caregivers. First, schools and social work agencies should offer training to primary caregivers on positive parenting techniques. This would help caregivers play a crucial mediating role between parents and children, especially in situations where parental discipline becomes ineffective due to physical distance. These skills can include setting boundaries, encouraging autonomy while maintaining oversight, and promoting constructive behavior. Second, given that primary caregivers often take a permissive or passive approach, agencies should provide guidance on how to implement more active supervision. This includes creating clear guidelines for smartphone use, monitoring children’s online activities, and addressing any negative behaviors without resorting to punitive or neglectful measures. Third, since parental discipline is often undermined by distance, it is vital to enhance communication channels. Schools and social work agencies can support primary caregivers by providing tools and resources to facilitate better communication between parents and children, even when physically separated. Encouraging regular check-ins or using technology to stay connected can help maintain a sense of parental involvement and guidance. Last, schools and social work agencies should also offer support and resources to primary caregivers on how to balance their caregiving roles with managing smartphone use. This includes understanding the implications of smartphone dependence, recognizing signs of excessive use, and taking proactive steps to reduce dependency through alternative activities, such as encouraging outdoor play or creative hobbies. Intervention strategies targeting primary caregivers can also facilitate the involvement of parents who are not physically present by offering online participation. This approach helps ensure consistency in parenting styles and strategies between parents and primary caregivers.

This study had some limitations. First, classifying smartphone dependence using a score threshold may lead to problematic outcomes, especially for children whose scores are near the cutoff. These children may be inaccurately categorized despite exhibiting signs of problematic use. Second, while csQCA is effective at identifying necessary and sufficient conditions as well as multiple pathways leading to an outcome, it is limited in capturing temporal dynamics and causal directionality. QCA is inherently cross-sectional and cannot easily establish temporal precedence, a critical element of causality in social science research. Therefore, future studies should incorporate complementary methodological approaches—such as longitudinal designs, process tracing, or mixed-method analyses—to strengthen causal inference and further validate our findings. Finally, the relatively small sample size of 20 cases may limit the robustness and generalizability of the findings. Future research with larger sample sizes is required to confirm and expand upon these findings.

## 6 Conclusion

In conclusion, this study employed QCA to investigate the formation mechanisms and adverse effects of smartphone dependence among rural children in China, focusing on the two key dimensions of children and their parents. Three pathways related to smartphone dependence were identified. The children who were not left behind, and who had poor self-control, insufficient supervision, and introverted personalities were more prone to smartphone dependence. Left-behind children in rural areas with poor self-control are likely to develop smartphone dependence, regardless of the quality of their parent–child relationship or personality type. Given the physical and mental immaturity of children, the findings led to the formulation of targeted intervention strategies from the perspectives of parents and primary caregivers.

## Data Availability

The original contributions presented in the study are included in the article/supplementary material, further inquiries can be directed to the corresponding author.
